# 1205. Antibiotic-prescribing for all respiratory tract diagnoses across a network of walk-in clinics: clinician-level variation and correlation with other performance metrics

**DOI:** 10.1093/ofid/ofad500.1045

**Published:** 2023-11-27

**Authors:** Daniel J Livorsi, Gosia Clore, Dilek Ince, Kelly M Percival, Amy O’Shea, Nathan Shaw, Kimberly Dukes, Stacey Hockett Sherlock, Eli Perencevich

**Affiliations:** University of Iowa Carver College of Medicine, Iowa City, Iowa; University of Iowa, Iowa City, Iowa; University of Iowa Hospitals & Clinics, Iowa City, Iowa; University of Iowa Hospitals & Clinics, Iowa City, Iowa; University of Iowa Carver College of Medicine, Iowa City, Iowa; University of Iowa Carver College of Medicine, Iowa City, Iowa; University of Iowa Carver College of Medicine, Iowa City, Iowa; University of Iowa Carver College of Medicine, Iowa City, Iowa; University of Iowa Carver College of Medicine, Iowa City, Iowa

## Abstract

**Background:**

It is unclear which stewardship metrics are most effective for audit and feedback to outpatient clinicians. In this study, we explored a metric that captures antibiotic-prescribing for all respiratory tract diagnoses to determine if it could serve as an appropriate metric for feedback.

**Methods:**

We performed a retrospective cohort study of in-person visits to the 7 walk-in clinics, including 3 Urgent Care locations, within University of Iowa Health Care during 2018-2022. Visits were categorized as respiratory visits (RVs) if a respiratory tract diagnosis was coded and the patient lacked complicating factors, such as a concomitant non-respiratory infection or certain comorbidities. We built a hierarchical logistic regression model that adjusted for antibiotic appropriateness tiers (1-3) to identify factors associated with antibiotic-prescribing for RVs. Using Spearman’s correlation, we compared the frequency at which clinicians prescribed antibiotics for RVs and other types of visits.

**Results:**

There were 331,496 visits, and 96,546 (29.1%) led to an antibiotic prescription; 44,498 (46.1%) of these were for RVs (Tables 1 and 2). At the clinician-level (n=89), the mean frequency of antibiotic-prescribing for RVs was 36.4% (standard deviation 11.6). Factors at the visit-level associated with an increased odds of antibiotic use for RVs included age ≥ 65 (OR=1.5; 95% CI=1.4-1.6) and having at least one comorbidity (OR=1.2; 95% CI=1.1-1.2). The frequency at which a clinician prescribed antibiotics for RVs was strongly correlated with antibiotic use for all visits (r=0.77, p< 0.001). Clinicians who more often prescribed antibiotics for RVs also more often prescribed antibiotics for respiratory infections that are always viral (r=0.73, p< 0.001), sinusitis (r=0.49, p< 0.001), and non-streptococcal pharyngitis (r=0.69, p< 0.001). There was no correlation between antibiotic-prescribing for RVs and the frequency of return visits within 30 days (r=0.11, p=0.29).

**Figure:**
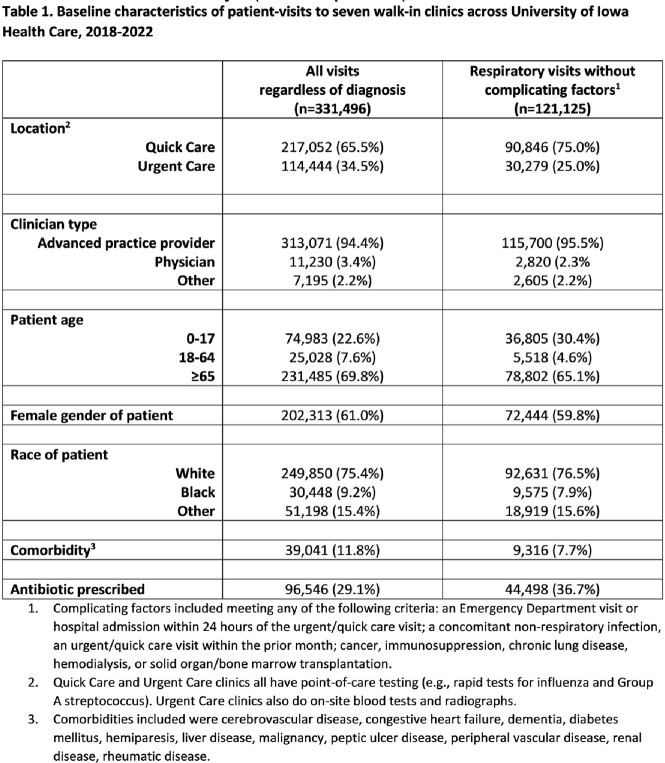

**Figure:**
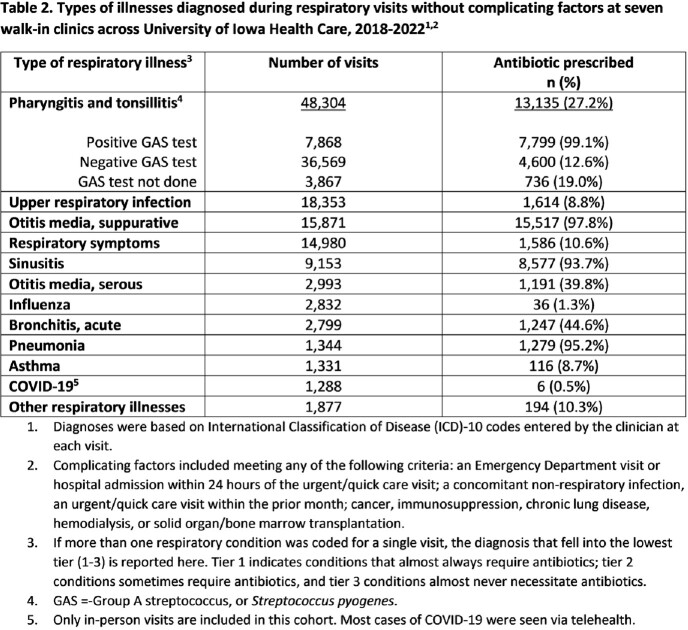

**Conclusion:**

A metric that quantifies the frequency of antibiotic-prescribing for all respiratory tract diagnoses correlated strongly with clinicians who over-use antibiotics for walk-in visits. Future studies should assess whether this type of metric is acceptable to clinicians and an effective tool for feedback.

**Disclosures:**

**Daniel J. Livorsi, MD**, Merck: Grant/Research Support **Kelly M. Percival, PharmD**, Gilead Sciences Inc: Advisor/Consultant

